# Electronic publication of new animal names - An interview with Frank-T. Krell, Commissioner of the International Commission on Zoological Nomenclature and Chair of the ICZN ZooBank Committee

**DOI:** 10.1186/1471-2148-12-184

**Published:** 2012-09-15

**Authors:** Frank-T Krell

**Affiliations:** 1 Department of Zoology, Denver Museum of Nature & Science, 2001 Colorado Boulevard, Denver, CO, 80205-5798, USA

## Abstract

On the 4th September 2012 the International Commission on Zoological Nomenclature announced an amendment to the International Code of Zoological Nomenclature allowing for electronic publication of the scientific names of animals. In this interview Frank-T. Krell discusses the implications of this amendment for authors wishing to publish descriptions of newly identified animal species in online and open access journals, and for the future of taxonomic science.

## 

Frank-Thorsten Krell, PhD, is the Curator of Entomology in the Department of Zoology, Denver Museum of Nature & Science, a Commissioner of the International Commission on Zoological Nomenclature and the Chair of the ICZN ZooBank Committee, responsible for implementing the official register for scientific animal names. He works on taxonomy and ecology of scarab beetles and is engaged in questions of zoological nomenclature and scientific publishing. He considers the recently published Amendment [1] to the International Code on Zoological Nomenclature [2] that allows electronic publication for nomenclatural purposes an important milestone in adapting the rules to dramatically changed working and publishing trends in the 21^st^ century.

## What was the problem with the previous Code?

Previously the International Commission on Zoological Nomenclature (ICZN), which is the body providing the rules on scientific animal names, did not recognise online-only journals. This meant that any animal name described in an online-only journal was not ‘available’ under the Code and would not be recognised as legally published. As an editor of "Systematic Entomology", a Wiley-Blackwell print and e-journal with electronic 'pre-publication,' I often felt the frustration of authors who were unsure whether they should allow electronic 'pre-publication' of their papers when the electronically published results remained unpublished under the Code and therefore ‘unavailable’ until the paper appeared in print at a later date. Authors who opted out of electronic pre-publication often had to wait over six months until their paper appeared in a printed issue. For authors publishing in open-access, online-only journals the situation was even more acute. Should they go for the widest dissemination of their results, but violate the Code? Some did [3]. The majority of taxonomists followed the rules, but then could not use open access journals. Over the years, the ICZN tried to find temporary solutions for online-only journals to publish in a manner compliant with the Code. Those e-journals who wanted to comply had to make available printed copies of those papers containing nomenclatural acts. But requiring such special treatment for taxonomic papers is unsustainable and not helpful to the reputation of taxonomy as a science.

## What has your role been in the process of getting the vote approved for electronic registration and publication of new species names?

As an active taxonomist and editor of scientific journals, I have constantly been exposed to the problems caused by the rule that declared electronic publications unavailable for the purposes of nomenclature, particularly describing new species or taxa. I have long championed [3,4] an amendment to the nomenclatural Code allowing electronic publications. As a Commissioner of the ICZN I chaired the session on electronic publication at a crucial meeting in Paris in 2008 [5]. At this meeting, we decided to propose an Amendment to the Code. It seemed overdue to adapt the rules in consideration of the increasing dominance of electronic publishing, thus better serving the needs of the zoological community. Many Commissioners were involved in the drafting process which was led by the Chair of the ICZN Editorial Committee, Gary Rosenberg, of the Academy of Natural Sciences of Drexel University in Philadelphia. As one of the initiators of an official register for scientific animal names [6] and Chair of the ICZN ZooBank Committee, I urged the Commission to require registration of electronically published names. In the final Amendment, we ended up requiring registration of electronically published works (i.e. the articles or books) and encourage registration of names. As my final involvement, I voted for the Amendment, as did a large majority of the Commissioners.

## How important is this for the field and how will it benefit zoological nomenclature in the future?

Most scientists, from physicists to taxonomists, receive and use their literature electronically, be it through subscriptions, interlibrary loan, or open access sources. For our everyday work, it is irrelevant whether a paper actually appears in print. The new Amendment will speed the process of publishing biodiversity information, improve access to this information through ZooBank, and can only help in reducing the ‘taxonomic impediment’ that hinders our discovery and cataloguing of zoological taxa.

## How does the new process work in comparison to the old method?

The old method still stands for print products. Print products needed and still need to fulfil certain criteria to be ‘available’ (i.e. comply with the Code) for nomenclatural purposes: Works must be in printed issues for the purpose of providing a public and permanent scientific record; an advertisement in a local newspaper would not count. Works must be obtainable, when first issued, free of charge or by purchase; a private publication distributed by the author or publisher to a handful of friends only would not count. Last but not least, it must have been produced in an edition containing simultaneously obtainable copies by a method that assures numerous identical and durable copies. However, it is a misconception that five copies of printed papers need to be deposited in libraries. Such a requirement never existed for print publications. The identical copies just needed to be obtainable. What has changed now? All the above criteria are still required for print publications, but additionally to "the numerous identical and durable copies", we are now allowing "widely accessible electronic copies with fixed content and layout". Those must still be issued for the permanent scientific record. A blog post that stays online for only a limited period of time would not count. Electronic publications have to fulfil a few additional criteria to be available: The date of publication must be stated in the work itself (i.e. the paper); the work (article or book) must be registered in ZooBank [7] and contain evidence that such registration has occurred; the registration entry must give the name and Internet address of an organization other than the publisher that is intended to permanently archive the work in a manner that preserves the content and layout, and is capable of doing so; the registration entry must also contain ISBN or ISSN of the registered work. All those requirements provide a safeguard that electronic works will remain retrievable in perpetuity. They do require some attention by publishers and authors, but compliance should be easy and painless. Publishers of electronic works now have the choice either to produce a paper edition of their works, or register and archive their electronic edition.

## How does this relate to open access journals and why is it so important for them?

Open access journals rarely produce print products. To publish nomenclaturally available papers under the old rules, open access publishers needed to make printed copies of those papers containing nomenclatural acts available. New names and nomenclatural acts were then classed as ‘available’ from this print run, but not from the original electronic publication. We had arrived at the awkward situation that open access papers, having the widest possible availability to readers, were unavailable for nomenclature. On the other hand, small print runs that hardly anybody can access were considered available. This situation contradicted Recommendation 8A of the Code: "Authors have a responsibility to ensure that new scientific names, nomenclatural acts, and information likely to affect nomenclature are made widely known". Now this is rectified. New species can be described in electronic format, and the paper is immediately available and can be used by taxonomists around the world. This makes open access journals even more attractive for authors of papers containing taxonomy, and helps disseminating taxonomic information faster and more widely.

## Why has it taken such a long time to get to this point given the popularity of the internet and open access publishing?

Every description of a new taxon, every nomenclatural act is a 'legal' document of lasting relevance [8]. Good taxonomic practice requires consulting the original publications. This is increasingly facilitated by electronic archives, such as the Biodiversity Heritage Library [9], or by open access publications such as this. However, the taxonomic community and the Commission are very much aware of the archival requirements necessary to maintain access in perpetuity to such publications. Paper has a proven track record as an archival medium. Electronic resources lack such a track record beyond a couple of decades. We all have experienced the technological evolution that rendered our old 5¼ inch floppy disks, and later their smaller cousins useless. Other media followed, or lost readability even before their reading machines were replaced. Old Word files or pdfs might become illegible over dozens of generations of new software versions. Every medium or format requiring a technical interface beyond optical lenses for human accessibility will be made redundant by technical progress. Despite the overwhelming acceptance of digital information for everyday work, their archival qualities are still widely doubted. When we first proposed the Amendment, we faced uncertainty or opposition amongst taxonomists [10], which we needed to assess and discuss. We are now confident that with registration in our new version of ZooBank and with the required intent to archive electronic works, we minimized the risk of information loss. The botanists' positive vote for electronic publication [11] is a further indication that we follow the right path.

## What do you think open access journals can do to help facilitate this process and smooth the transition?

Open access journals have a crucial role in proving the concepts of electronic publishing and registration in taxonomy. They demonstrate how to make taxonomic information widely and freely available which is particularly advantageous for our colleagues in biodiversity-rich but financially challenged countries.

Since open access journals normally have no paper edition, they need to deal with registration and archiving according to the new rules. To smooth the registration process, minimize man-made errors (typos), and to reduce costs in terms of time and salaries, we need to implement automated registration. We will develop a system that allows publishers to bulk-upload marked-up content into ZooBank. Several journals, both open access and traditional, have expressed interest in working with us on such a solution. Registration needs to be painless and efficient. If too much work is involved, if it is too cumbersome, it won't be widely accepted. GenBank has been a success story because journal editors became convinced of its usefulness and made submission of sequences to the database a condition for publication [12]. The mycological community follows the same route with several journals requiring registration of new names in MycoBank [13]. A few journals already require registration of new zoological names in ZooBank, e.g., ZooKeys and PLoS ONE. BMC journals will soon follow. If registration is quick and easy, we are confident that other journals will join in, even if they produce a paper edition and are not required to register.

## What are the next steps and what does the future hold?

With electronic publication being a new concept in zoological nomenclature, we need to see how it works out. The Amendment will serve as a test run. The ICZN has started on a new edition of the Code that will contain the regulations of the Amendment in principle. Experience with the Amendment will help us to fine-tune the rules for the new Code in the future.

We will also continue to develop ZooBank, add further modules allowing registration of other nomenclatural acts beyond new names, defining and publishing our policies, improving the registration process for all parties, and working out a procedure for validation or verification of registered information.

## Where does this lead to?

Wouldn't it be great to have one authoritative place to access all information about all animal species ever published? We will not achieve this with ZooBank alone, but after some time of data gathering, all scientific animal names, all nomenclatural acts, and all original descriptions will be documented in ZooBank. We will not achieve this ambitious goal overnight, but I am confident that we will achieve it. My vision always has been to have all original descriptions either stored or linked to in ZooBank: a “One Stop Shop” for everything related to animal names. When registration will have become common practice and widely accepted as an integral part of the taxonomic publication process, then we can consider making it a requirement for availability. ZooBank would then be a complete registry. When and where was a species described? How many species were described in this genus? Did I overlook a species for my generic revision? The answers will be all in one place. This is easy to achieve with open access publications and archives. But with a large portion of taxonomic research being done without funding, be it by amateurs [14] or on the side by professionals, it is unlikely that all taxonomic research will be published open-access in the near future. A significant part of taxonomic research will continue to only be accessible behind the pay-wall of subscriptions. However, early during the development of ZooBank, several commercial publishers have already signalled that they will allow ZooBank to make original descriptions freely available. We might achieve the One Stop Shop for animal names after all.

## Competing interests

Frank Krell is Commissioner of the International Commission on Zoological Nomenclature and Editor-in-Chief of the Denver Museum of Nature & Science Annals, an open access journal.

## Author’s contributions

FTK wrote and approved the final text.
